# An Atypical Presentation of Reticular Erythematous Mucinosis: A Case Report and Comprehensive Literature Review

**DOI:** 10.3390/jcm14062131

**Published:** 2025-03-20

**Authors:** Beatrice Bălăceanu-Gurău, Cristina Violeta Tutunaru, Olguța Anca Orzan

**Affiliations:** 1 Faculty of Medicine, “Carol Davila” University of Medicine and Pharmacy, 020021 Bucharest, Romania; beatrice.balaceanu@drd.umfcd.ro (B.B.-G.); olguta.orzan@umfcd.ro (O.A.O.); 2 Department of Oncologic Dermatology, “Elias” Emergency University Hospital, 011461 Bucharest, Romania; 3 Department of Dermatology, Faculty of Medicine, University of Medicine and Pharmacy of Craiova, 200349 Craiova, Romania

**Keywords:** cutaneous mucinosis, reticular erythematous mucinosis, dermatopathology, mucin, dermoscopy

## Abstract

Reticular erythematous mucinosis (REM) is a rare form of primary cutaneous mucinosis, often linked to viral infections, inflammatory conditions, ultraviolet radiation, radiotherapy, malignant disorders, or an underlying immune dysfunction. It typically affects middle-aged women and manifests as symmetrical erythematous macules, papules, or plaques that exhibit a reticular and annular configuration, mainly on the midline of the thorax or dorsum. Although these regions represent the most prevalent sites, atypical occurrences have been noted. We report an unusual case of REM in a pediatric female patient with an ongoing history of B-cell acute lymphoblastic leukemia. The physical examination revealed an atypical distribution of REM lesions, symmetrically affecting the gluteal region and proximal thighs. Establishing a definitive diagnosis required a meticulous correlation between clinical, dermoscopic, and histopathologic findings. To our knowledge, this is the first documented case of REM in a patient with acute lymphoblastic leukemia. Our study underlines the importance of including REM in the differential diagnosis of persistent erythematous lesions, particularly in immunocompromised patients or those with a history of malignancy. Furthermore, we provide a comprehensive literature review, emphasizing the etiology, risk factors, pathogenetic mechanisms, diagnostic challenges, and different therapeutic options for REM.

## 1. Introduction

Cutaneous mucinosis encompasses a diverse group of disorders defined by localized or widespread accumulation of mucin within the dermis [1]. Reticular erythematous mucinosis (REM) is a chronic and diffuse variant of primary cutaneous mucinosis, also referred to as plaque-like cutaneous mucinosis [1,2,3,4]. REM can occur in patients of all ages and both sexes, although it is most commonly observed in middle-aged women [5,6,7]. REM is infrequently encountered before the age of 10 or after 60 [5,6,7,8]. Most cases are sporadic [5].

Despite its unknown etiology, reports of familial occurrences involving siblings (brother and sister as well as twins, respectively) suggest a potential genetic predisposition [1,6,9,10,11,12]. Although, Caputo et al. identified polymorphisms in HLA types in a familial case of REM, it remains unclear whether any specific HLA allele confers susceptibility to REM, as the limited number of documented cases and inconsistent HLA typing preclude definitive conclusions [12].

To accurately diagnose REM, clinicians must differentiate it from other conditions through clinical, histopathological, and laboratory correlation, particularly lupus erythematosus tumidus (LET), papular mucinosis (PM), lichen myxedematosus (LM), dermatomyositis (DM), scleredema, confluent and reticulated papillomatosis of Gougerot-Carteaud, prurigo pigmentosa, disseminated granuloma annulare, papular sarcoidosis, papular mucinosis, and mucopolysaccharidosis [1,4].

Clinically, REM presents with a spectrum of cutaneous manifestations such as erythematous macules, papules, or plaques that merge into a reticular and annular configuration, predominantly affecting the midline of the chest or back and has generally a symmetrical distribution [1,2,3,5,6,13,14]. Beyond the predilection sites, cases of REM have been documented in atypical locations, including the face, lower extremities, upper extremities, abdomen, and surgical scars [2,4,7,13,15,16]. For instance, Kenny et al. reported a case of localized REM-like eruption on the lower legs that mimicked cutaneous larva migrans [13]. Notably, these atypical presentations are usually accompanied by concurrent lesions on the midline [2]. Mucous membranes, genitalia, and internal organs are generally not affected [6]. Although typically asymptomatic, pruritus is reported in approximately 30% of cases [1,4,5,7]. Neither serologic nor systemic abnormalities are present [3,4,17]. REM typically follows a cyclic clinical course characterized by periods of remission and exacerbation [6,7,13,18].

Dermoscopic studies on REM have also been conducted, providing valuable insights into its vascular and structural patterns. Betancourch et al. revealed the presence of linear blood vessels over translucent globular yellowish areas without a defined structure or scaling collar, resembling an apple jelly pattern, which may assist in the differential diagnosis when compared to other clinically similar entities [19,20]. Another study by Takada et al. revealed two main features: dotted vessels and uniform structureless yellowish-white spots and patches [1]. The authors concluded that dermoscopy may enhance clinical evaluation and aid in distinguishing REM from LET, PM, DM, and scleredema [1].

Histologically, REM is characterized by mild perivascular lymphocytic infiltrates in the superficial and mid-dermis, often accompanied by mast cells, histiocytes, and factor XIIIa-positive dendrocytes [1,4,6,21]. Additional findings may include perifollicular infiltration, slight vascular dilation, and focal hemorrhage in the papillary dermis [1,4,6,21]. A hallmark feature is the separation of dermal collagen bundles with basophilic mucin deposits, primarily surrounding infiltrates, appendages, and the upper dermis; furthermore, occasional stellate cells may also be noted [1,4]. The epidermis is typically unaffected, though mild spongiosis, lichenoid changes, basal layer degeneration, and elastic fiber fragmentation can occur [1,4,6]. Colloidal iron staining reliably detects mucin, outperforming Alcian blue, which may yield false negatives [4]. Direct immunofluorescence is generally negative, though granular IgM and C3 deposits at the dermo–epidermal junction may be seen in a minority of cases [3,4,6,7,22]. Electron microscopy reveals expanded intercollagenous spaces, elastic fiber fragmentation, activated fibroblasts, and tubular inclusions in various cell types, including endothelial cells and dermal macrophages [1,4].

In this review, we describe an unusual case of REM in a B-cell acute lymphoblastic leukemia teenager female patient with an atypical distribution of lesions. Furthermore, we provide a comprehensive review of the literature, emphasizing the etiology, risk factors, pathogenetic mechanisms, and diagnostic challenges, warranting careful consideration in the differential diagnosis of recurrent or persistent erythematous lesions, particularly in patients with a history of malignancies. Additionally, we discuss different therapeutic approaches, highlighting their efficacy and limitations.

## 2. Case Report

We report the case of a 13-year-old female patient with Fitzpatrick skin type III, slightly overweight (with a body mass index of 26), who presented to the Clinical Dermatology Department in Craiova, Romania, in January 2024, with an asymptomatic cutaneous eruption that began three months earlier on the elbows and progressively spread to other areas. She has a history of beta-thalassemia and was diagnosed with B-cell acute lymphoblastic leukemia in 2017. The patient underwent chemotherapy following the BFM protocol from 2017 to 2020, achieving complete remission, with multiple pediatric hospitalizations during this period. Since June 2022, she has been on chelation therapy for iron metabolism disorders.

**Figure 1:**
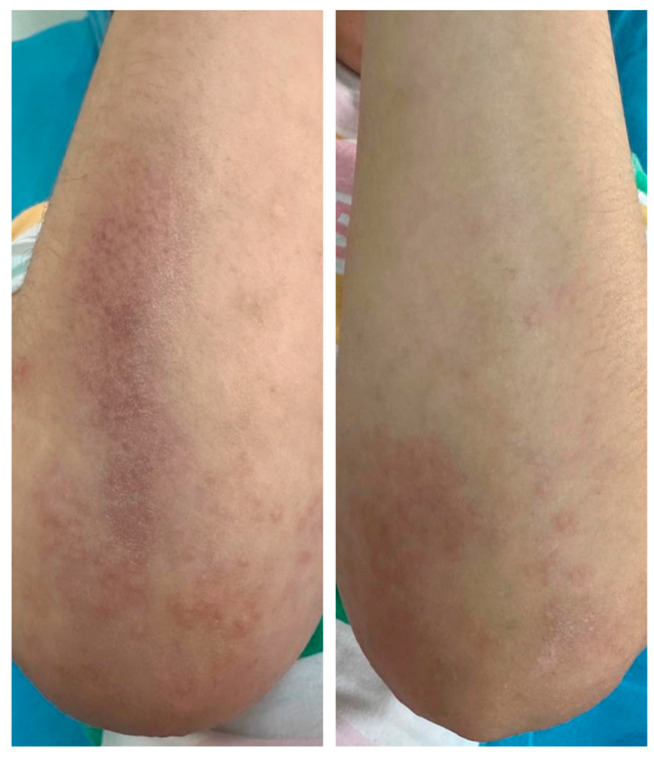
Macular and papular erythematous lesions, slightly indurated, confluent into plaques and large patches, displaying a reticular pattern, symmetrically distributed on the extensor surfaces of the forearms and elbows.

**Figure 2:**
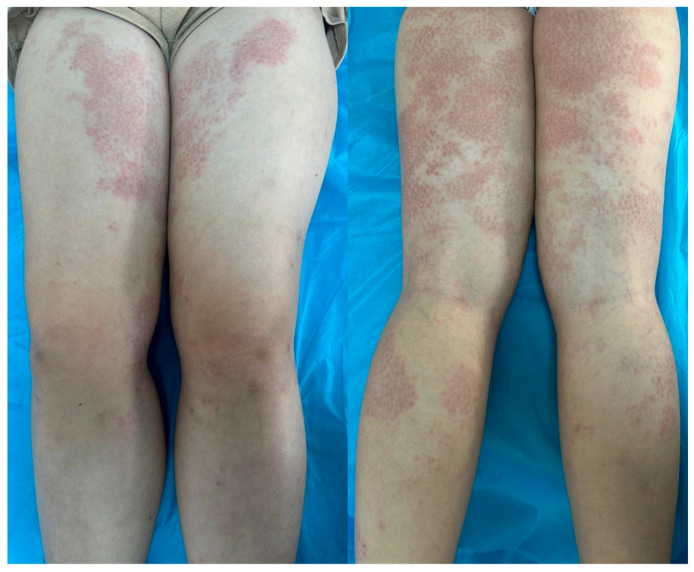
Macular and papular erythematous lesions, slightly indurated, confluent into plaques and large patches, displaying a reticular pattern, symmetrically distributed at the level of the thighs, extending towards the upper third of the shins.

**Figure 3:**
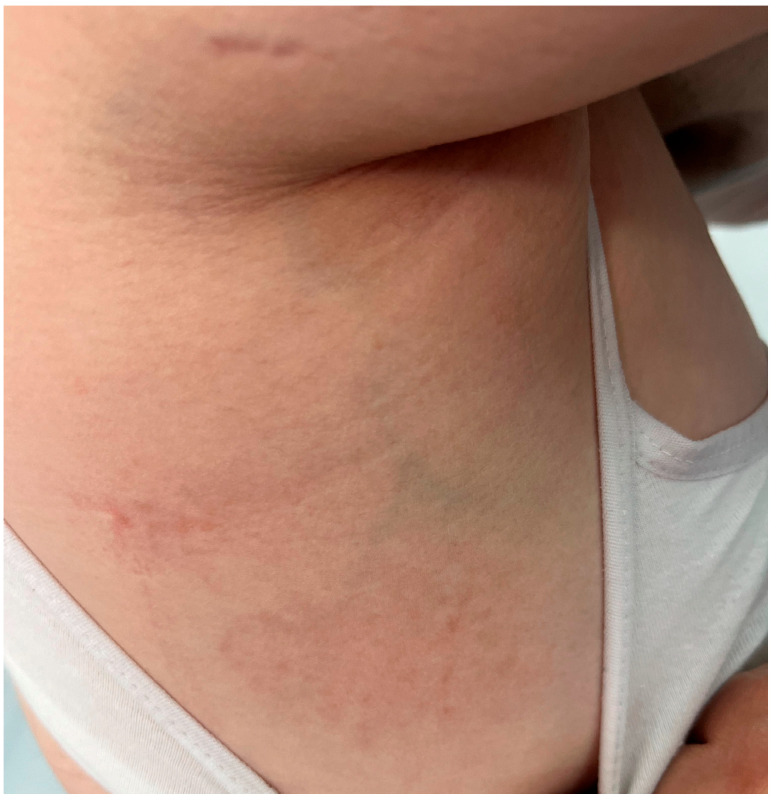
Macular and papular erythematous lesions, slightly indurated, confluent into plaques and large patches, displaying a reticular pattern, symmetrically distributed at the level of the right lateral thoracic region.

Physical examination revealed symmetrically distributed erythematous macules and papules, slightly indurated and coalescing into plaques and large patches with a reticular pattern (Figure 1, Figure 2 and Figure 3). These lesions were primarily located on the buttocks and thighs, extending to the upper third of the shins, and were also observed on the extensor surfaces of the forearms, elbows, and the right lateral thoracic region (Figure 1, Figure 2 and Figure 3).

Laboratory tests at admission revealed a mildly reduced hemoglobin level of 10.9 g/dL (normal range: 12.0–16.0 g/dL) and a hematocrit of 32.8% (normal range: 36–46%). Additionally, the mean corpuscular volume was decreased, recorded as being at 60 fL (normal range: 80–100 fL), and the mean corpuscular hemoglobin was low, recorded at 17 pg (normal range: 27–33 pg). Ferritin level was elevated, recorded at 234.8 ng/mL (normal: 15–150 ng/mL). Thyroid hormones (TSH, T3 and T4) antinuclear antibodies, immunoglobulin levels (IgA, IgG, IgM), thyroid antibodies (anti-microsomal, anti-peroxidase, and antithyroglobulin), antinuclear antibody (ANA), double-stranded DNA (dsDNA) antibody, Sjögren’s syndrome A (SSA/Ro) and B (SSB/La) antibodies, scleroderma 70 (Scl−70) antibody, and serum complement were within normal ranges. Antibodies to native single- and double-stranded DNA were negative.

**Figure 4:**
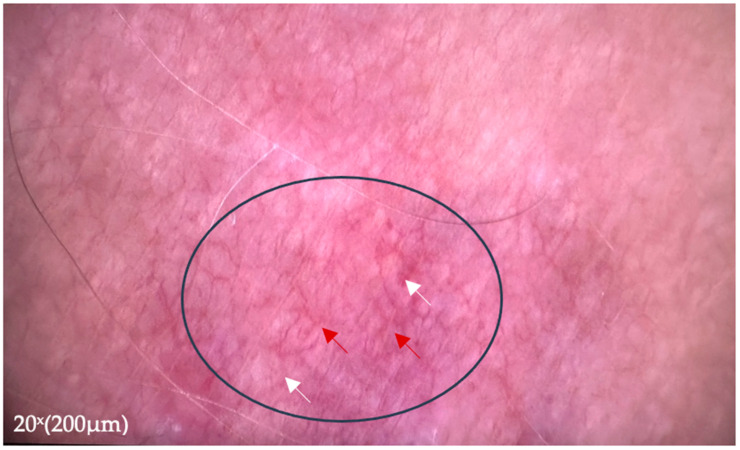
Dermoscopic examination of one lesion located on the right tight revealing irregular blood vessels (red arrows) over translucent globular yellowish area (white arrows). Image was captured using a higher-magnification digital dermoscope (20×), corresponding to a 200 µm scale bar.

Dermoscopic examination revealed irregular vessels over yellowish-white areas (Figure 4).

**Figure 5:**
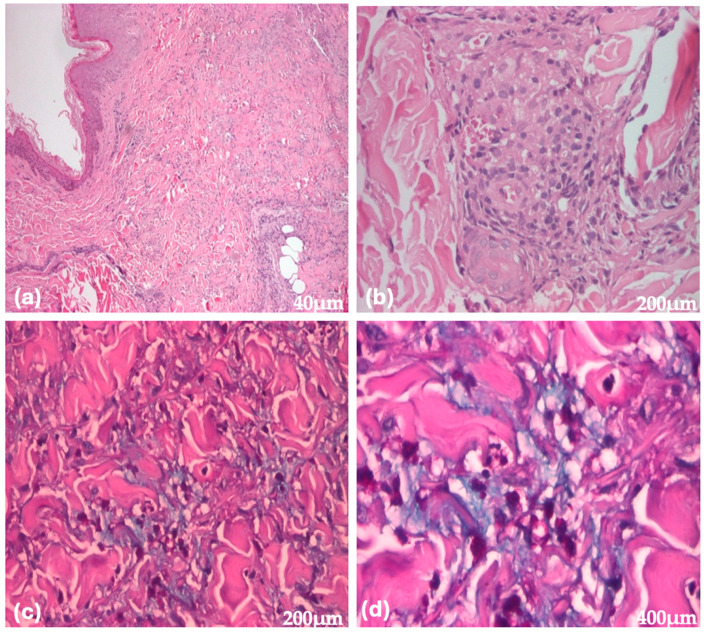
Histopathological images of REM stained with hematoxylin and eosin (H&E) and Alcian blue (**a**–**d**). Images show hyper-orthokeratosis and vacuolization of some keratinocytes (**a**,**b**). In the dermis, there are capillary-type vessels with stasis, leukodiapedesis, and a perivascular lymphohistiocytic inflammatory infiltrate; small areas of AB/PAS positivity can be observed among the collagen bands in the reticular dermis (**c**,**d**).

A punch skin biopsy was performed due to clinical suspicion of cutaneous mucinosis, and special stains for mucin were requested. Histopathological examination of the skin biopsy showed hyper-orthokeratosis and vacuolization of some keratinocytes (Figure 5). The dermis exhibited capillary-type vessels with stasis, leukodiapedesis, and a perivascular lymphohistiocytic inflammatory infiltrate (Figure 5). Alcian blue/Periodic Acid-Schiff (AB/PAS) staining demonstrated small areas of AB/PAS positivity among the collagen bands in the reticular dermis. Based on the clinical presentation and histopathological findings, a diagnosis of REM was established.

**Figure 6:**
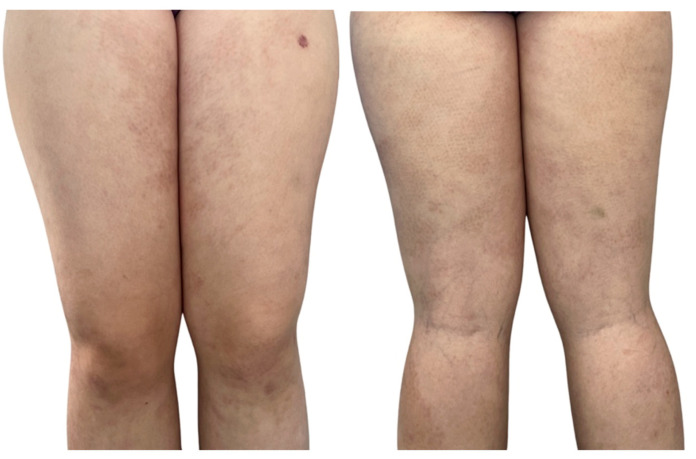
Clinical evolution after 6 weeks showing a favorable response to 32 mg of methylprednisolone daily.

Considering the extent of the lesions, systemic corticosteroid therapy was initiated with methylprednisolone at 32 mg per day, accompanied by a proton pump inhibitor. Antimalarial therapy was initially avoided due to the patient’s age and hematologic history. After six weeks, the patient showed a favorable response with a marked reduction in erythema intensity, although complete resolution of the lesions was not achieved. Side effects included increased appetite, mild facial edema, and a worsening of depressive symptoms. Consequently, the methylprednisolone dosage was reduced to 16 mg per day for two weeks, followed by the introduction of hydroxychloroquine at 200 mg/day after normal ophthalmological examination. The dose was then reduced to once daily for two months, but only a fair response was observed and she discontinued the treatment due to side effects. A significant clinical improvement was observed at the 3-month follow-up visit (Figure 6).

To our knowledge this is the first reported case of REM associated with acute lymphoblastic leukemia. However, a direct association with REM cannot be established. A limitation of this case is the absence of cytokine analysis and CD4/CD8 immunostaining, which could have provided further insights into the immunopathogenesis of REM. These investigations are not part of the standard diagnostic workup and not essential for diagnosing REM, although cytokine profiling might have helped elucidate the role of inflammatory mediators such as interleukin 1β (IL−1β), IL−6, tumor necrosis factor α (TNF-α), and TGF-β in mucin metabolism and fibroblast activation. Likewise, CD4/CD8 immunostaining could have contributed to a better understanding of the immune profile of the lesions and their distinction from other inflammatory dermatoses.

## 3. Discussion

### 3.1. Overview

REM is an uncommon and insufficiently elucidated medical condition, with merely around 100 instances documented in the literature in English [4,5,8,11]. The classification of this disease is subject to ongoing debate owing to its overlapping characteristics with other mucinoses and autoimmune disorders. The potential triggers, pathogenesis, and clinical course exhibit considerable variability, thereby complicating its identification and therapeutic management. Various factors, including ultraviolet (UV) radiation, radiotherapy, hormonal changes (such as menstruation, pregnancy, and oral contraceptive use), immunological dysfunctions, viral infections (HIV), and Borrelia infections, have been linked to REM’s pathogenesis [1,4,5,6,11,23].

### 3.2. Potential Triggers and Associations of REM

Photobiological studies by Adamski et al. demonstrated that full-body ultraviolet A1 (UVA1) irradiation could induce REM both clinically and histologically, unlike isolated ultraviolet B (UVB) or UVA exposure [18]. This suggests that additional elements like heat and perspiration may contribute to lesion development [18]. Viral involvement has also been considered, based on viral-like inclusions found in lesional endothelial cells, though these may also result from elevated interferon levels, as seen in lupus erythematosus (LEs) [4]. Tenea et al. reported the first documented case of REM in an HIV-positive African patient, underlining the need for diagnostic focus in immunocompromised individuals [5].

The potential link between REM and inflammatory or autoimmune disorders is not well elucidated, although consistent associations have been observed with Hashimoto’s thyroiditis, hyperthyroidism, uveitis, systemic lupus erythematosus (SLE)/LET, discoid lupus erythematosus, diabetes mellitus, myxedema, paraproteinemia, myopathy, polyneuropathy, and idiopathic thrombocytopenic purpura [1,2,3,5,6,7,17]. Interestingly, REM may precede systemic lupus erythematosus (SLEs), as noted by Del Pozo et al., who observed SLEs developing years after REM onset [24].

The literature concerning REM in individuals diagnosed with lung, breast, and colon malignancies indicates a plausible association with various neoplasms [2,4,6,8]. The most pronounced correlation has been identified in patients with lung cancer, potentially due to the involvement of transforming growth factor-β, ILs, TBF, and interferon, which exhibit elevated levels in alveolar macrophages among individuals afflicted with lung cancer [2,4,16]. Overexpression of MUC1 and MUC4 in lung carcinomas may further contribute to REM pathogenesis [4,25]. Caputo et al. revealed the first documented REM cases mimicking mycosis fungoides, which were unusually aggressive and resistant to standard therapies [4,26]. Additionally, REM has been noted at mastectomy sites and in abdominal flaps post-mammary reconstruction, reinforcing its association with malignancy and tissue trauma [11,15].

### 3.3. REM Pathogenesis: Hyaluronic Acid Deposition and Immune Dysregulation

Although research studies on REM have explored the nature of mucin deposition, the triggers for its increased production, and its clinical significance, the exact mechanism underlying glycosaminoglycan deposition, particularly hyaluronic acid (HA), remains unclear [3,5]. It is hypothesized that an abnormal fibroblast response to certain cytokines, particularly IL−1β, may contribute to the dysregulated hyaluronic acid metabolism associated with REM [1,3,4,5,7,21]. UVA1 radiation, either directly or indirectly by inducing proinflammatory cytokines such as IL−1, can stimulate matrix-degrading enzymes like proteoglycans in dermal fibroblasts, leading to enhanced HA degradation [3]. These cytokines may also reduce HA synthesis by fibroblasts in REM [3]. Additionally, reactive oxygen species (ROS) produced by UV exposure, particularly singlet oxygen, superoxide anion, and hydroxyl radicals generated by UVA, contribute to HA fragmentation [27].

In REM skin lesions, mucin deposits primarily consist of HA, as revealed by glycosaminoglycan analysis [27]. A study by Tominanga et al. demonstrated that HA levels in REM lesions were approximately 2.9 times higher than in uninvolved skin, although fibroblasts from the lesions did not show altered synthetic activity [21]. Immunohistochemical analysis using anti-factor XIIIa (anti-FXIIIa) antibodies revealed a significantly increased number of FXIIIa+ cells in lesional skin compared to uninvolved and normal control skin [21]. Since hyaluronan is synthesized by hyaluronic acid synthase (HAS), the study also investigated the expression of HAS isoforms (HAS1, HAS2, and HAS3) and found a significant increase in HAS2+ cells within affected skin (*p* < 0.01) [21]. Notably, the HAS2 antibody stained several populations of FXIIIa+ cells in REM lesions, suggesting that the hyaluronan accumulation is linked to factor XIIIa+/HAS2+ dermal dendrocytes rather than dermal fibroblasts [21]. HA overexpression is observed in the early (edematous, inflammatory) stages of REM, whereas fibroblasts in later-stage lesions may lose this expression, suggesting that fibroblasts exhibit different phenotypes at various disease stages [5].

REM has not been extensively characterized from an immunological perspective. Some studies have reported elevated circulating immune complex (CIC) levels in patients with REM during the initial presentation and recurrences [5]. In the case report from Tenea et al., normal complement and CIC levels were maintained throughout a 6-year follow-up period [5]. Additionally, REM has been associated with significantly reduced natural killer (NK) cell cytolytic activity, indicating potential disruptions in immunoregulatory pathways [5]. This immunological alteration may contribute to the observed predisposition to autoimmunity, neoplasms, and endocrine disorders in REM patients [5].

### 3.4. Differential Diagnosis

Accurate diagnosis of REM requires clinicians to distinguish it from other conditions through a combination of clinical evaluation, histopathological analysis, and laboratory correlation (Table 1). The main differential diagnoses include LET, PM, LM, DM, scleredema, confluent and reticulated papillomatosis of Gougerot-Carteaud, prurigo pigmentosa, disseminated granuloma annulare, papular sarcoidosis, papular mucinosis, and mucopolysaccharidosis (Table 1) [1,4,19]. Other differential diagnoses include Jessner’s lymphocytic infiltration (JLI), palpable arciform migratory erythema (PAME), polymorphic light eruption, and erythema annulare centrifugum (Table 1) [11,18]. Histologically, these conditions may be indistinguishable from REM, highlighting the importance of clinicopathologic correlation [11,18].

Some researchers suggest that REM is a variant of LET due to overlapping clinical and histopathological features, UV-induced flare-ups, and a favorable response to systemic antimalarial therapy (Table 2) [1,2,3,6,28]. Regarding the clinical presentation, in the case of REM, erythematous macules and papules arranged in a reticulated pattern are observed over the midline of the anterior neck, chest, and back in sun-protected areas [1,2,6]. This pattern contrasts with LET, which typically presents as erythematous to violaceous plaques or nodules, often in annular patterns on sun-exposed sites [1,6]. Both conditions share a predominance in females [4].

Regardless of the overlapping features, histopathological differences support the classification of REM as a distinct entity separate from LET (Table 2) [5,24,29]. Despite the presence of perivascular and perifollicular T-lymphocytic infiltration, with an increased CD4/CD8 ratio, the absence of interface dermatitis, the deposition of dermal mucin, the potential presence of plasmacytoid dendritic cells (PDCs), and negative immunofluorescence staining, there are notable histopathological differences between the two entities [4,5,7,24,29]. REM is characterized by a less dense and more superficial infiltrate, shallower mucin deposition, and less frequent immunoglobulin and complement deposits along the dermo–epidermal junction (DEJ) [29]. In REM, minimal epidermal changes with subtle pigment incontinence are observed, whereas LET exhibits more pronounced epidermal involvement, including atrophy, focal basal vacuolar changes, and a thickened basal membrane [5]. Mucin deposition, highlighted by Alcian blue staining, is typically localized to the superficial and mid-reticular dermis in REM, while it extends to deeper dermal layers in LET [5,7,14]. The inflammatory infiltrate in REM, primarily composed of T-helper lymphocytes, is sparse and confined to the superficial interstitial and perivascular dermis [5]. In contrast, LET displays a more extensive infiltrate that extends into the deeper dermis and demonstrates a predilection for hair follicles, in addition to the perivascular distribution [5]. While both REM and LET demonstrate PDCs within the infiltrate, LET shows a higher percentage of positive cases and a greater number of PDC clusters, occasionally with peri-adnexal distribution and deeper involvement [29]. The relevance of these differences remains uncertain but may be related to the topographic distribution of the presumptive etiologic agent or differential activation of receptors such as toll-like receptors (TLR)-7 or TLR9 [29]. DIF findings with granular deposits of IgM and C3 along the DEJ suggest that immunoglobulins and their complements play a more significant role in tissue damage in LET compared to REM [4,5,24,29].

**Table 2:** Differentiating REM from LET: clinical presentation and histopathological characteristics.

Feature	REM	LET	References
Proposed Relationship	Considered a variant of LET due to overlapping clinical and histopathological features, UV-induced flare-ups, and response to antimalarial therapy.	Shares features but considered a distinct entity due to specific histopathological differences.	[1,2,3,6,27]
Clinical Presentation	Erythematous macules and papules in a reticulated pattern on the midline of the anterior neck, chest, and back in sun-protected areas.	Erythematous to violaceous plaques or nodules, often in annular patterns on sun-exposed sites.	[1,2,6]
Gender Predominance	Female predominance.	Female predominance.	[4]
Histopathological Overview	Less dense and superficial infiltrate; shallow mucin deposition; less frequent Ig and complement deposits along the dermo–epidermal junction.	Denser infiltrate extending into the deeper dermis with frequent immunoglobulin and complement deposits along the dermo–epidermal junction.	[10,20,28]
Epidermal Changes	Minimal changes with subtle pigment incontinence.	More pronounced involvement, including atrophy, focal basal vacuolar changes, and thickened basal membrane.	[10]
Mucin Deposition	Localized to the superficial and mid-reticular dermis.	Extends to deeper dermal layers.	[10,12,15]
Inflammatory Infiltrate	Sparse, superficial, mainly T-helper lymphocytes in the interstitial and perivascular dermis.	Extensive, deeper infiltrate with a predilection for hair follicles.	[10]
Plasmacytoid dendritic cells	Present but in lower frequency and in clusters.	Higher percentages and clusters, sometimes peri-adnexal and deeper.	[28]
Direct immunofluorescence findings	Negative or less frequent granular deposits of IgM and C3 along the dermo–epidermal junction.	More frequent granular deposits of IgM and C3 along the dermo–epidermal junction, indicating a more significant role of immunoglobulins and complement in tissue damage.	[4,10,20,28]

DM affects sun-exposed areas and is not confined to the trunk [1,11]. It is typically characterized by the appearance of erythematous to violaceous papules and plaques, which are symmetrically distributed over the extensor areas of the metacarpophalangeal and interphalangeal joints or by the appearance of a heliotrope eruption on the upper eyelids [1,11]. Although dermal mucin deposition can also occur in DM, the absence of epidermal atrophy, hyperkeratosis, and basal cell vacuolar changes in REM helps to distinguish the two conditions from a histopathologic point of view [11].

Scleredema is characterized by firm, woody plaques, and diffuse, symmetric, non-pitting skin induration, primarily affecting the upper back, posterior neck, and face [1,4]. LM presents as firm, waxy, normochromic or erythematous papules ranging from 1 to 4 mm, typically arranged symmetrically on the hands, fingers, arms, face, upper torso, and legs [1]. Seborrheic dermatitis and tinea versicolor can affect the central chest, but they are typically accompanied by scaling, facilitating clinical differentiation [4]. Similarly, confluent and reticulated papillomatosis presents with characteristic scaling, aiding its distinction from REM [4]. Generalized myxedema is associated with hypothyroidism and presents with mild skin thickening around the eyes, nose, cheeks, and distal extremities [4]. Pretibial myxedema, an uncommon manifestation of Graves’ disease, is characterized by non-pitting edema and thickening on the anterior lower legs [4].

### 3.5. Therapeutic Approaches

Notably, REM is often self-limiting and may resolve spontaneously, even after prolonged durations [1,4]. REM generally responds more favorably to treatment compared to other types of mucinoses [3]. The initial management of REM includes sun avoidance and photoprotection [1]. There have been scientific reports of REM skin lesions resolving following colon cancer treatment and thyroid hormone replacement therapy, even in the absence of thyroid function abnormalities [8,11]. Table 3 lists various therapeutic approaches.

#### 3.5.1. Antimalarial Agents

Treatment with antimalarial agents has demonstrated effectiveness in resolving lesions in most cases, significantly reducing disease duration [4,5,30]. Hydroxychloroquine, administered in doses of 200–400 mg daily, is the first-line treatment for REM and has proved to result in significant improvements in skin lesions, due to its impact on circulating immune complexes [1,3,4,5,6,7,31]. Discontinuation can cause a rebound in immune complexes and disease recurrence [4,32]. Other conventional antimalarial agents such as chloroquine are also considered effective therapeutic options [1,3,5,6,7]. In 2011, Kreuter et al. reported a case series involving 11 patients with REM treated with either chloroquine or hydroxychloroquine [33]. The median age was 44 years, with the majority being smokers (91% [10/11]) and having autoimmune disorders (55% [6/11]), predominantly thyroid disease [33]. Significant clinical improvement was observed after 3 and 12 months of treatment (*p* < 0.001) [33].

Due to dose-related toxicity, baseline ophthalmologic evaluation is required, followed by regular follow-up every 6 to 12 months [7]. Quinacrine has been proposed as an alternative therapy for patients with allergies or ocular contraindications [1,34]. However, in cases unresponsive to systemic antimalarials or where contraindications exist, alternative therapies have been proposed, including topical and systemic corticosteroids, cyclosporine, calcineurin inhibitors, colchicine, oral antihistamines, tetracycline, pulsed dye laser (PDL), and a 308 nm excimer laser, as well as UVA and UVB phototherapy [3,6,19,27,35,36,37,38].

#### 3.5.2. Calcineurin Inhibitors

Pimecrolimus, an immunomodulating macrolactam with potent anti-inflammatory properties and minimal systemic immunosuppression, has shown promise in managing REM [4,6]. It inhibits T-cell activation and reduces the synthesis of inflammatory cytokines [4,6]. Rubegni et al. reported successful management with tacrolimus, an immunosuppressant that inhibits T-cell activity by blocking calcineurin [35,39].

#### 3.5.3. Pulsed Dye Laser (PDL)

In REM syndrome, a PDL has been histologically shown to reduce mucin deposition and lymphocytic infiltration [6]. Although the exact mechanism remains unclear, it is thought to damage small blood vessels by means of selective photothermolysis and therefore activate immunological processes [4,6,36]. It is generally safe with minimal side effects [4,36]. Use of a PDL has demonstrated efficacy in the treatment of REM, as evidenced by Greve et al., who reported near-complete remission in two female patients with minimal adverse effects, primarily localized hypopigmentation [4,36]. Additionally, a comparative study by Mansouri et al. highlighted the therapeutic benefit of pimecrolimus in conjunction with a PDL [6]. In this case, a woman with REM on the chest, upper back, and arms underwent treatment with twice-daily 1% pimecrolimus cream for five months, supplemented by two PDL sessions for symmetrical lesions, leading to near-complete clinical resolution [6]. These findings underscore the potential of PDLs, either alone or in combination with immunomodulatory agents, as an effective therapeutic option for REM [6].

#### 3.5.4. Phototherapy

Paradoxically, REM lesions have shown improvement with solar exposure and phototherapy, despite the known role of UV radiation in exacerbating certain inflammatory dermatoses [3,31,40]. Among the available phototherapeutic options, UVA1 radiation has been particularly effective due to its deep dermal penetration and lower risk of erythema and cellular transformation compared to other wavelengths [40]. UVA1 exerts its effects by inducing apoptosis in T and B lymphocytes and immature proliferating mast cells, thereby modulating immune responses [40]. Additionally, it stimulates dermal fibroblasts to produce matrix-degrading enzymes, including proteoglycans, via IL−1β induction, leading to accelerated hyaluronic acid degradation [4]. Furthermore, reactive oxygen species (ROS) generated by UVA exposure contribute to HA breakdown, facilitating lesion resolution [4]. The efficacy of UVA1 as a treatment for REM was further demonstrated by Ahmerd-Hoekstra et al., while Meewes et al. reported complete remission of lesions in a middle-aged woman after 18 sessions of UVA1 irradiation, totaling a cumulative dose of 1210 J/cm² [27,40].

In addition to UVA1, 308 nm excimer laser UVB therapy has been investigated as a potential alternative [37]. Myioshi et al. observed its promising role in REM treatment, although the precise mechanism remains unclear [37]. Recent studies suggest that UVB reduces hyaluronic acid levels in the papillary dermis by downregulating HAS1, HAS2, and HAS3 mRNA expression [37,41]. Moreover, UVB exposure has been shown to decrease transforming growth factor-β1 (TGF-β1) and TGF-β1-receptor II expression, both of which play roles in HA metabolism and fibroblast activity [37,41]. While conventional UVB has limited dermal penetration, targeted monochromatic 308 nm excimer light appears to exert direct and indirect effects on dermal lesions, making it a promising therapeutic option [37,41].

Despite these advancements, alternative therapeutic approaches continue to be explored, with varying degrees of success [5]. In recalcitrant cases, dapsone has been proposed as a potential treatment due to its anti-inflammatory properties and reactive oxygen species (ROS) scavenging effects [42]. Dapsone, widely used in chronic inflammatory dermatoses such as dermatitis herpetiformis and prurigo pigmentosa, has also shown potential as an adjuvant therapy in LES [4,42]. However, its use in REM remains off-label, as no controlled clinical trials have been conducted to confirm its efficacy [4].

**Table 3:** Therapeutic approaches for REM.

Treatment Option	Effectiveness and Outcomes	Considerations and Side Effects	References
Hydroxychloroquine	First-line treatment; reduces lesions by affecting immune complexes.	Risk of retinal damage; needs eye check-ups.	[1,3,4,5,6,7,31]
Chloroquine	Effective in some cases.	Similar risks as hydroxychloroquine.	[1,3,5,6,7,33]
Quinacrine	Alternative for those who cannot take hydroxychloroquine.	Fewer eye-related side effects.	[1,34]
Topical/Systemic Corticosteroids	Reduces inflammation and redness.	Long-term use may cause side effects.	[3,6,19,27,35,36,37,38]
Tacrolimus	Reduces inflammation.	Can cause local irritation.	[35,39]
Pimecrolimus	Decreases inflammation and cytokines.	Skin redness.	[4,6]
Pulsed Dye Laser	Reduces redness and mucin deposits.	May cause temporary light spots; generally safe.	[4,6,36]
UVA1 Phototherapy	Effective in resistant cases.	Fewer side effects than other phototherapy types.	[3,31,40]
308 nm Excimer Laser	Promising for hard-to-treat cases.	Local irritation; good for targeted areas.	[37,41]
Dapsone	Helpful in tough cases (anti-inflammatory).	Off-label use; limited clinical trial evidence.	[4,42]
Combination Therapy (e.g., Pimecrolimus+ Pulsed Dye Laser)	Near-complete resolution in some cases.	Combining treatments enhances the effectiveness.	[6]

## 4. Conclusions

As research continues to explore the underlying mechanisms and effective treatments for REM, the clinical and histopathological overlap with LT warrants further examination, potentially enhancing the understanding of both conditions. Our interesting and uncommon case represented a diagnostic challenge. Factors including the patient’s age (13-year-old teenage girl) and the distribution of the lesions (predominantly on the legs) are not typically representative of classic REM. Establishing a definitive diagnosis required a meticulous correlation between dermatologic and histopathologic findings. This case emphasizes the need to integrate clinical and pathological assessments in dermatology, particularly when encountering atypical presentations. Studies like this contribute to dermatologists’ knowledge and expertise while continuing medical education programs can improve their ability to recognize and manage REM effectively.

## Figures and Tables

**Table 1:** Differential diagnoses of REM with key distinguishing features.

Differential Diagnosis	Distinguishing Feature	References
Lupus Erythematosus Tumidus	Sun-exposed areas, IgM/C3 deposits on DIF, deeper mucin deposition.	[1,3,6,10,14,28]
Dermatomyositis	Heliotrope rash, Gottron’s papules, muscle weakness, perifascicular atrophy.	[1,11]
Scleredema	Firm, woody plaques, and non-pitting skin induration affecting upper back and neck.	[1,4]
Lichen Myxedematosus	Waxy, firm papules are symmetrically distributed on hands, arms, face, and torso.	[1]
Mycosis Fungoides	Patch/plaque-stage lymphoma, T-cell clonal proliferation, CD4+ predominance.	[4,26]
Seborrheic Dermatitis	Scaling, greasy appearance. Favors sebaceous areas (scalp, central chest).	[4]
Tinea Versicolor	Hypopigmented or hyperpigmented patches with fine scaling. Potassium hydroxide (KOH) positive.	[4]
Jessner’s Lymphocytic Infiltration	Recurrent erythematous plaques, absent mucin deposition, lymphocytic infiltrate	[11,18]
Prurigo Pigmentosa	Sudden onset erythematous reticulated plaques. Histologically distinct.	[11,18]
Erythema Annulare Centrifugum	Annular erythematous lesions with a trailing scale, and a self-limiting course.	[11,18]

## Data Availability

This review summarizes data reported in the literature and it does not report primary data.
